# Targeting the cancer-associated fibroblasts as a treatment in triple-negative breast cancer

**DOI:** 10.18632/oncotarget.12658

**Published:** 2016-10-14

**Authors:** Ken Takai, Annie Le, Valerie M. Weaver, Zena Werb

**Affiliations:** ^1^ Department of Anatomy, University of California, San Francisco, CA, USA; ^2^ Department of Surgery and Center for Bioengineering and Tissue Regeneration, University of California, San Francisco, CA, USA; ^3^ Present address: Division of Breast Oncology, Saitama Cancer Center, Saitama, Japan; ^4^ Present address: St. George's University School of Medicine, Grenada

**Keywords:** pirfenidone, triple-negative breast cancer, fibrosis, cancer-associated fibroblast, transforming growth factor-β

## Abstract

Increased collagen expression in tumors is associated with increased risk of metastasis, and triple-negative breast cancer (TNBC) has the highest propensity to develop distant metastases when there is evidence of central fibrosis. Transforming growth factor-β (TGF-β) ligands regulated by cancer-associated fibroblasts (CAFs) promote accumulation of fibrosis and cancer progression. In the present study, we have evaluated TNBC tumors with enhanced collagen to determine whether we can reduce metastasis by targeting the CAFs with Pirfenidone (PFD), an anti-fibrotic agent as well as a TGF-β antagonist. In patient-derived xenograft models, TNBC tumors exhibited accumulated collagen and activated TGF-β signaling, and developed lung metastasis. Next, primary CAFs were established from 4T1 TNBC homograft tumors, TNBC xenograft tumors and tumor specimens of breast cancer patients. CAFs promoted primary tumor growth with more fibrosis and TGF-β activation and lung metastasis in 4T1 mouse model. We then examined the effects of PFD *in vitro* and *in vivo*. We found that PFD had inhibitory effects on cell viability and collagen production of CAFs in 2D culture. Furthermore, CAFs enhanced tumor growth and PFD inhibited the tumor growth induced by CAFs by causing apoptosis in the 3D co-culture assay of 4T1 tumor cells and CAFs. *In vivo*, PFD alone inhibited tumor fibrosis and TGF-β signaling but did not inhibit tumor growth and lung metastasis. However, PFD inhibited tumor growth and lung metastasis synergistically in combination with doxorubicin. Thus, PFD has great potential for a novel clinically applicable TNBC therapy that targets tumor-stromal interaction.

## INTRODUCTION

Collagen, mainly produced by fibroblasts, is the most abundant extracellular matrix (ECM) protein in the stroma. Collagen metabolism is deregulated in many chronic diseases including cancer [1]. Increased type I collagen expression and desmoplasia/fibrosis in tumors are associated with increased risk of metastasis [2–4]. An increasing body of evidence suggests that activated cancer-associated fibroblasts (CAFs) can promote cancer fibrosis and progression [1, 5–10]. Transforming growth factor-β (TGF-β) ligands produced by cancer cells and/or CAFs promote the accumulation of fibrotic desmoplastic tissue and the rate of cancer progression [1, 10, 11].

Breast cancer is the most commonly diagnosed cancer among women and the second-most frequent cause of cancer death. Of the various classes of human breast cancer, triple-negative (ER^−^PR^−^HER2^−^) breast cancer (TNBC) is the most aggressive type, and no targeted therapy is available. In addition, TNBC has the highest propensity to develop distant metastases and show poor prognosis when there is evidence of central fibrosis [12]. TGF-β ligands are often enriched in the TNBC tumor microenvironment [13–16]. This suggests that targeting the desmoplasia/fibrosis and TGF-β signaling in TNBC could be of value.

In the present study, we have evaluated TNBC tumors that have enhanced collagen expression to determine whether we can reduce metastasis by targeting the CAFs with Pirfenidone (PFD). PFD is an orally administered pyridine (5-methyl-1-phenyl-2-[1H]-pyridone) that exhibits antifibrotic properties in a variety of *in vitro* and animal models of fibrosis as a TGF-β antagonist, and has been clinically developed for the treatment of idiopathic pulmonary fibrosis (IPF) [17, 18].

## RESULTS

### TNBC xenograft tumors exhibit accumulated collagen and activated TGF-β signaling, and metastasize to lungs

**Figure 1:**
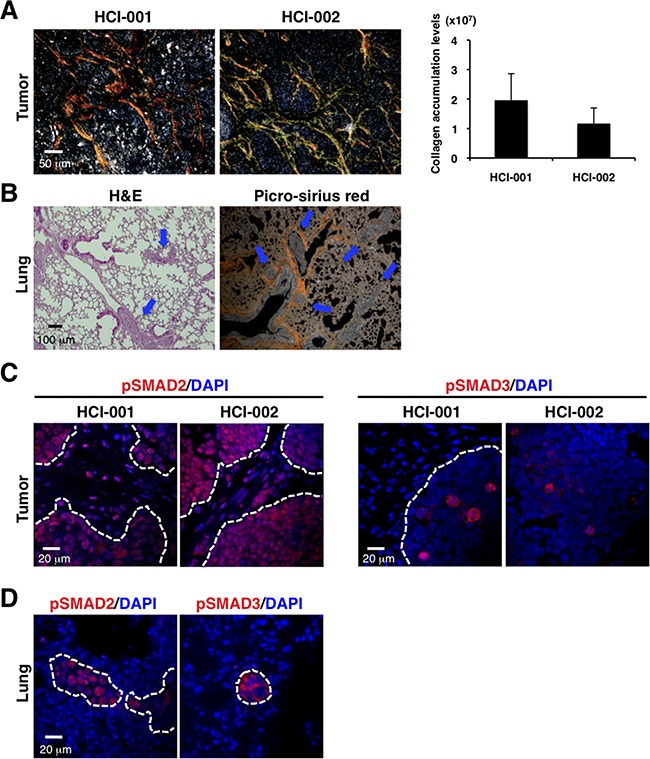
PDX models of TNBC exhibit enhanced collagen accumulation, activated TGF-β signaling and lung metastasis **A.** TNBC xenograft tumors show enhanced collagen accumulation by picro-sirius red staining (left panel). Fibrillar collagen was quantified by picro-sirius red staining using ImageJ software. n=2-3 (right panel). **B.** Lung metastasis was detected in the TNBC xenograft model by H&E staining (left panel) and picro-sirius red staining (right panel). **C.** TNBC xenograft tumors were immunostained with anti-phospho-SMAD2 (red on left panel) and anti-phospho-SMAD3 (red on right panel) antibodies. DAPI (blue) stained nuclei. Phospho-SMAD2 was widely expressed in primary tumors and stroma. Phospho-SMAD3 was sporadically expressed in primary tumors, not in stroma. **D.** Lungs of the TNBC xenograft model were immunostained with anti-phospho-SMAD2 (red on left panel) and anti-phospho-SMAD3 (red on right panel) antibodies. DAPI (blue) stained nuclei. Phospho-SMAD2 and phospho-SMAD3 were expressed in the lung metastatic tumors, but not the stroma around the small tumors.

To determine fibrosis and TGF-β activation in TNBC as a model, we used patient-derived xenograft (PDX) models that retain the essential features of the original patient tumors and metastasis to specific sites (HCI-001 and HCI-002) [19, 20], and thus are authentic experimental systems for studying human cancer metastasis. In these models, the tumors engrafted in the mammary glands of immunodeficient NOD/SCID mice grew to approximately 1 cm in 5-8 weeks (Supplementary Figure S1A). Enhanced collagen accumulation was exhibited in the primary tumors (Figure 1A). Lung metastasis was also detected in HCI-001 model (Figure 1B and Supplementary Figure S1B). Although some reports show collagen deposition in metastatic sites of mice and patients [21, 22], we did not detect marked collagen accumulation in the lung metastases (Figure 1B, right panel).

To evaluate TGF-β signaling, we determined expression of phospho-SMAD2 and phospho-SMAD3 as intracellular markers of TGF-β signaling [23–25]. Phospho-SMAD2 was widely expressed in primary tumors and stroma, while phospho-SMAD3 was sporadically expressed in primary tumors, but not in stroma (Figure 1C). Phospho-SMAD2 and phospho-SMAD3 were expressed in the lung metastatic tumors and also in the stroma around the large metastases, but not in the stroma around the micrometastases (Figure 1D and Supplementary Figure S1D). These observations of our TNBC xenograft models are consistent with TNBC-related fibrosis and TGF-β signaling showed previously [12–16].

### CAFs promote primary tumor growth and lung metastasis in TNBC mouse model

**Figure 2:**
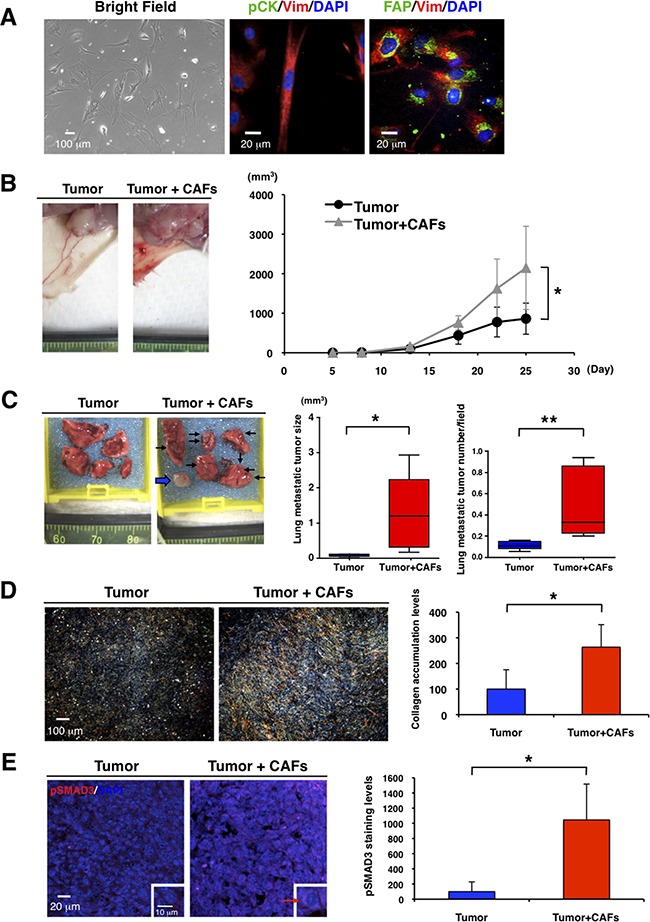
CAFs promote primary tumor growth and lung metastasis in TNBC mouse model **A.** We cultured CAFs from fresh tumor specimens of breast cancer patients in a 3% O_2_ incubator (Bright Field, left panel). Immunofluorescence of cultured CAFs was conducted by using anti-vimentin (Vim, red), anti-pan-cytokeratin (pCK, green in middle panel), and anti-fibroblast activation protein (FAP, green in right panel) antibodies. DAPI (blue) stained nuclei. CAFs were stained with anti-vimentin and anti-FAP antibodies. **B.** We transplanted 4T1 cells (1x10^4^) without or with CAFs (2x10^4^) into mammary glands of BALB/c mice (n=5). Representative photographs of 4T1 primary tumor after transplantation with or without the CAFs are shown (left panel). CAFs promoted primary tumor growth. Tumor volume (mm^3^) was measured by “V=0.52xW^2^xL.” W=width (mm), L=length (mm). *p<0.02 (right panel). **C.** Representative photographs of lungs showed that CAFs promoted lung metastasis. Arrows indicate visible lung metastatic tumors and a broad arrow indicates a metastatic lymph node in the right brachium (left panel). Volume (mm^3^) of single metastatic tumor was measured by “V=0.52xW^2^xL.” W=width (mm), L=length (mm). *p<0.05 (middle panel). H&E staining showed that CAFs increased lung metastatic tumor number (*p<0.02) (right panel). n=5. **D.** Representative photographs of picro-sirius red staining showed that CAFs promoted primary tumor fibrosis (left panel). Collagen deposition marked by picro-sirius red staining was quantified by using ImageJ software. CAFs enhanced collagen accumulation in primary tumors. n=5, *p<0.01 (right panel). **E.** Primary tumors were immunostained with an anti-phospho-SMAD3 antibody (red). DAPI (blue) stained nuclei. Representative photographs showed that CAFs enhanced expression level of phospho-SMAD3 in primary tumors (left panel). Expression levels of phospho-SMAD3 in primary tumors were quantified by using ImageJ software. n=3, *p<0.02 (right panel).

CAFs have been reported to promote breast tumor progression *in vitro* and *in vivo*, although it has yet to be determined whether normal mammary fibroblasts suppress or promote breast cancers [26–29]. We isolated CAFs from tumor specimens of luminal-type breast cancer patients (Figure 2A, left panel). Vimentin and fibroblast activation protein (FAP) are markers commonly used for identification of CAFs, as FAP is not expressed in adult normal tissue [1, 6, 10, 29, 30]. The cultured cells had an elongated appearance and reduced cell-cell contact and expressed vimentin and the CAF marker FAP, but not pan-cytokeratin, an epithelial tumor marker (Figure 2A, middle and right panels and Supplementary Figure S2A and S2B). We then cultured CAFs derived from the TNBC xenograft tumors (Supplementary Figure S2C). Since the CAFs did not express human-specific vimentin (Supplementary Figure S2D), these data suggest that the dominant fibroblast population in the xenograft models is derived from the mouse.

We next determined the effects of CAFs on TNBC *in vivo*. We cultured CAFs derived from 4T1, a mouse TNBC cell line, homograft tumors (Supplementary Figure S2E and MATERIALS AND METHODS). When we transplanted 4T1 cells with or without those CAFs into mammary glands of BALB/c mice, CAFs promoted primary tumor growth (Figure 2B) and increased lung metastatic tumor size and numbers (Figure 2C). Significantly, CAFs enhanced collagen accumulation and increased the expression levels of phospho-SMAD3 in primary tumors (Figure 2D and 2E). These results suggest that CAFs may enhance TNBC progression through TGF-β activation, as described previously [1, 10, 11].

### PFD has inhibitory effects on cell viability and collagen production in CAFs

**Figure 3:**
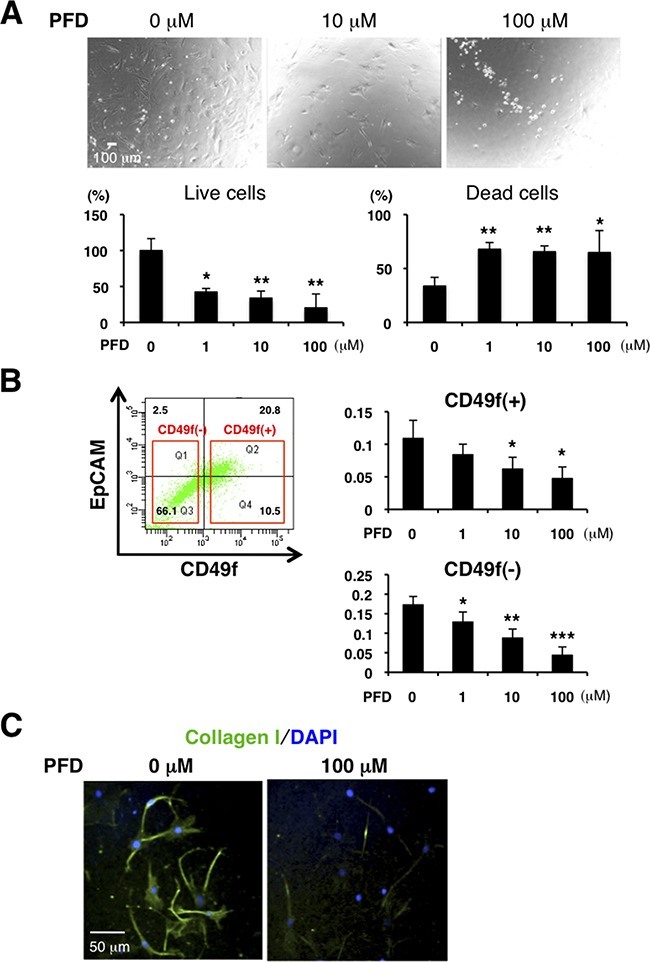
PFD has inhibitory effects on cell viability and collagen production in CAFs **A.** We cultured CAFs derived from tumor specimens of luminal-type breast cancer patients in a 3% O_2_ incubator and treated them with PFD in triplicate. Representative photographs are shown (upper panels). Total cells were stained with trypan blue on Day 4. Live cells decreased and dead cells increased with higher concentration of PFD (lower panels). *p<0.05, **p<0.01 compared to the control condition. **B.** FACS analysis was conducted from dissociated HCI-001 TNBC xenograft tumor cells by using anti-CD49f and anti-EpCAM antibodies and then CD49f^+^ cells (tumor cells) and CD49f^−^ cells (mainly stromal cells) were sorted. 4,000 CD49f^+^ cells or 10,000 CD49f^−^ cells were plated with each PFD concentration for culture and MTT assay was performed on day 15. Cell viability of CD49f^+^ and CD49f^−^ cells decreased with higher concentration of PFD. *p<0.05, **p<0.01, ***p<0.001 compared to the control conditions. **C.** We cultured CAFs derived from HCI-002 TNBC xenograft tumors. Immunofluorescence of the cultured CAFs used an anti-collagen I (green) antibody. DAPI (blue) stained nuclei. Collagen production was decreased by PFD.

Our data from the PDX tumors indicate that some TNBC have increased fibrosis and TGF-β. We therefore hypothesized that an anti-fibrotic agent as well as a TGF-β antagonist may be effective for TNBC treatment. PFD exhibits antifibrotic properties in a variety of in vitro and animal models of fibrosis [31–35], and has shown efficacy and safety in patients with liver fibrosis, renal fibrosis and idiopathic pulmonary fibrosis (IPF) [36–40]. In animal models of fibrosis in the lung, liver, kidney and heart, PFD reduces fibrosis and downregulates TGF-β and other molecules [33, 41–45]. We first evaluated toxicity of PFD in normal mammary organoids by 3D assay and confirmed PFD was not toxic at 100μM (Supplementary Figure S3A). According to previous reports, higher concentrations than those in our tests do not cause death of normal fibroblasts [31, 33]. However, PFD decreased the number of live CAFs and increased the number of dead CAFs from tumor specimens of luminal-type breast cancer patients (Figure 3A).

PFD inhibited growth of mouse CAFs isolated from the TNBC xenograft tumors in 2D culture (Supplementary Figure S3B). We then dissociated TNBC xenografts and sorted them into CD49f^+^ (tumor) and CD49f^−^ (mainly stroma) cell population by flow cytometry. Cell viability of both CD49f^+^ and CD49f^−^ cells, as measured by the MTT assay, decreased with increasing concentrations of PFD (Figure 3B). Since those cells did not proliferate in the assay, this result indicates that PFD promotes cell death. In addition, PFD inhibited collagen production by mouse CAFs (Figure 3C). These results show that PFD is an effective regulator of both CAF viability and collagen production in culture.

### PFD inhibits TNBC growth induced by CAFs

**Figure 4:**
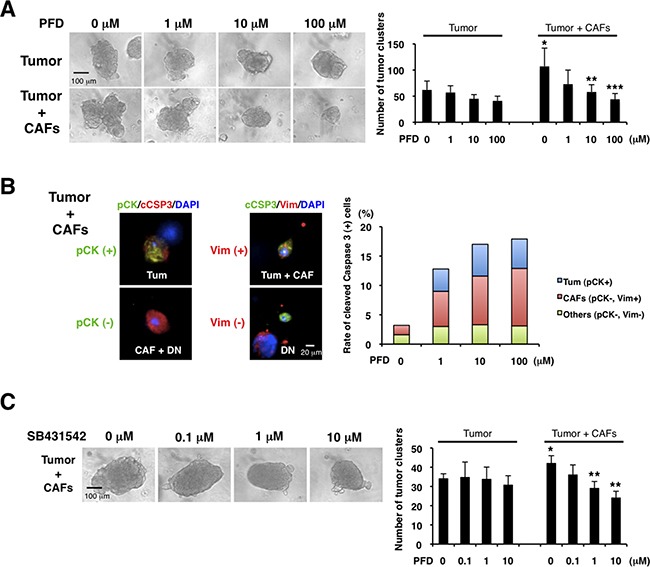
Pirfenidone inhibits TNBC growth induced by CAFs We cultured CAFs derived from TNBC xenograft tumors (HCI-001), and 3D co-culture assayed the CAFs and aggregated 4T1 cells (tumor cluster) in Matrigel. **A.** We examined the effects of PFD (triplicate). CAFs increased the tumor cluster size and PFD inhibited the size increased by CAFs (left panel). Also, CAFs increased the number of tumor clusters and PFD decreased the tumor cluster number increased by CAFs (right panel). *p<0.05 compared to tumor (0 μM). **p<0.05 or ***p<0.02 compared to 0 μM (Tumor + CAFs). **B.** We conducted immunofluorescence of the 3D Matrigel cultures by using anti-pan-cytokeratin (pCK, green, left panel), anti-vimentin (Vim, red, middle panel) and cleaved caspase-3 (cCSP3, red, left and green, middle panels, respectively) antibodies. DAPI (blue) stained nuclei. 4T1 tumor cells expressed both pan-cytokeratin and vimentin, and CAFs expressed vimentin. Therefore, pan-cytokeratin^+^ cells were 4T1 cells, and vimentin^+^ cells were either 4T1 cells or CAFs. Double-negative cells were regarded as other cell type. We counted the numbers of those cells (except 4T1 tumor clusters) with cleaved caspase-3 (representative photographs in left and middle panels) and quantified each apoptotic cells (right panel). We found that PFD induced apoptosis of 4T1 tumor cells and CAFs. **C.** We examined the effects of a TGF-β inhibitor (SB431542) in the 3D co-culture assay (triplicate). PFD decreased the tumor cluster size (left panel) and the tumor cluster number (right panel). *p<0.05 compared to tumor (0 μM). **p<0.01 compared to 0 μM (Tumor + CAFs).

Since CAFs can promote cancer progression [1, 7–10], we examined the effects of PFD on tumor-stromal interaction in 3D co-culture assays of the mouse CAFs (see Supplementary Figure S2C) and 4T1 cells. We observed that CAFs enhanced tumor growth. Interestingly, PFD had little inhibitory effect on the 4T1 tumor without CAFs, but strongly inhibited the tumor growth induced by CAFs (Figure 4A). We then characterized the nature of the inhibition by immunofluorescence of tumor cells and CAFs in the 3D co-culture. Using phospho-histone H3 and cleaved caspase-3 antibodies we observed that PFD induced apoptosis of both tumor and CAFs (Figure 4B), but did not inhibit tumor cell mitosis (Supplementary Figure S4).

Since TGF-β is important for the tumor-stromal interaction [1, 10, 11, 46] and PFD can inhibit TGF-β [33, 41–45], we hypothesized that TGF-β inhibition by PFD is the mechanism of the suppressive interaction. We found that a specific TGF-β inhibitor, SB431542, inhibited the tumor growth induced by CAFs, but not growth without CAFs in the same 3D co-culture assay (Figure 4C). Those findings suggest that PFD inhibits TNBC growth by targeting TGF-β in tumor-stromal interaction.

### PFD inhibits primary tumor growth and lung metastasis in combination with doxorubicin in TNBC mouse model

**Figure 5:**
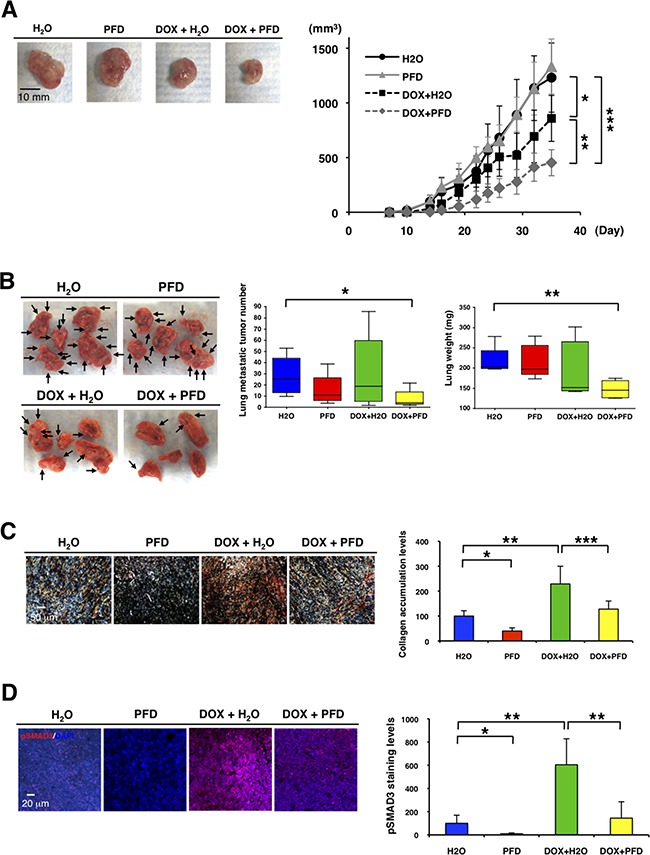
Pirfenidone inhibits primary tumor growth and lung metastasis in combination with doxorubicin in TNBC mouse model We transplanted 4T1 cells (1x10^4^) and 4T1-stimulated CAFs (2x10^4^) into mammary glands of BALB/c mice (n=5-6). PFD (50 mg/kg) or water was orally administered two times per day and doxorubicin (4 mg/kg) or PBS was injected from the mouse tail vain on day 0 and 19. **A.** Representative photographs of primary tumors after the treatments (Day 37) (left panel). Tumor volume (mm^3^) was measured by “V=0.52xW^2^xL.” W=width (mm), L=length (mm). PFD had no effect on primary tumor growth, but inhibited the tumor growth synergistically in combination with doxorubicin. *p<0.05, **p<0.01, ***p<0.001 (right panel). **B.** Representative photographs of lung after the treatments (Day 37) (left panel). Visible lung metastatic tumor numbers in five lobes were counted. PFD decreased the lung metastatic tumor numbers in combination with doxorubicin, but PFD monotherapy did not decrease the numbers. *p<0.02 (middle panel). Total lung weight was measured. PFD decreased tumor weight in combination with doxorubicin though PFD monotherapy did not decrease the weight. **p<0.002 (right panel). **C.** Representative photographs of primary tumors by picro-sirius red staining showed that doxorubicin enhanced and PFD inhibited collagen accumulation in primary tumors (left panel). Collagen deposition visualized by picro-sirius red staining was quantified by using ImageJ software. n=3, *p<0.01, **p<0.02, ***p<0.05 (right panel). **D.** Primary tumors were immunostained with an anti-phospho-SMAD3 antibody (red). DAPI (blue) stained nuclei (left panel). Expression levels of phospho-SMAD3 were quantified by using ImageJ software. Doxorubicin enhanced and PFD inhibited phospho-SMAD3 levels in primary tumors. n=4, *p<0.02, **p<0.01 (right panel).

We next tested the effects of PFD on TNBC *in vivo* using the 4T1 mouse model. Prior to *in vivo* experiments, we verified that CAFs from 4T1 homograft tumors enhanced tumor growth, while PFD inhibited the tumor growth induced by the CAFs in 3D co-culture assay (Supplementary Figure S5A). We then transplanted 4T1 cells and CAFs into the mammary glands of BALB/c mice. We administered PFD (50 mg/kg) or water orally two times per day. We also tested the interaction of PFD treatment with chemotherapy by injecting doxorubicin (4 mg/kg) in the tail vein on days 0 and 19. While PFD alone did not inhibit the primary tumor growth (Figure 5A), doxorubicin alone inhibited primary tumor growth, and PFD together with doxorubicin inhibited tumor growth synergistically (Figure 5A). PFD or doxorubicin alone did not reduce the number of lung metastatic tumors (Figure 5B). However, PFD in combination with doxorubicin inhibited lung metastasis significantly (Figure 5B). In a second experimental protocol, we administered an increased concentration of PFD (100 mg/kg) two times per day in combination with doxorubicin and found an even more marked decrease in lung metastatic tumor numbers and weight compared to control (Supplementary Figure S5B).

We next determined the effect of PFD inhibition of CAFs on apoptosis, collagen accumulation and phospho-SMAD3 expression in the primary tumors. Although treatment of the mice with PFD had no effect on apoptosis in α-SMA^+^ CAFs and tumor cells (data not shown), it decreased CAFs significantly (Supplementary Figure S5C). Treatment of the mice with doxorubicin enhanced collagen accumulation (Figure 5C). However, treatment of the mice with PFD or PFD plus doxorubicin inhibited collagen accumulation significantly (Figure 5C). Phospho-SMAD3 expression levels paralleled the collagen accumulation levels (Figure 5D). Therefore, we suggest that simultaneous inhibition of TGF-β by PFD along with chemotherapy with doxorubicin may overcome the activation of TGF-β and enhance therapeutic effects of treatment for TNBC. Taken together, our findings indicate that downregulating TGF-β signaling pathway with PFD in combination with doxorubicin can inhibit tumor-stromal interaction, collagen accumulation and suppress TNBC progression.

## DISCUSSION

The importance of the microenvironment for the response to cancer therapy is an emerging field. While many studies have targeted angiogenesis and inflammation/immune function, several investigations recently have focused on stromal collagenous extracellular matrix and CAFs as potential targets [27, 47–58]. In this study, we showed that PFD inhibited tumor growth of TNBC *in vitro* by targeting CAFs. *In vivo*, PFD inhibited the tumor growth and lung metastasis synergistically in combination with doxorubicin.

We observed that mouse CAFs promoted tumor progression *in vitro* and *in vivo* as previously reported [1, 6–10]. This occurred in both PDX models in NOD/SCID immunosuppressed mice and 4T1 tumors in immunosufficient Balb/C mice. TGF-β signaling was activated both in the tumor cells and the stroma of the xenograft tumors (Figure 1C). This is in keeping with previous studies showing that TGF-β signaling in fibroblasts is important to promote tumor growth [46]. Since 4T1 cells express high levels of TGF-β [59], it is likely that tumor-induced TGF-β promoted transformation of mouse fibroblasts into CAFs *in vivo*. This hypothesis is supported by our finding that PFD, which inhibits TGF-β, promoted cell death and suppressed collagen production in cultured CAFs, as seen previously in *in vitro* studies of fibroblasts [31–35].

Our 3D co-culture assays show that PFD strongly inhibited tumor growth promoted by CAFs but had little inhibitory effect on tumor growth without CAFs. These findings suggest that PFD inhibits TNBC growth more effectively by targeting tumor-stromal interaction than by targeting the tumor itself. PDGF-A and HGF are also reported to be molecular targets of PFD for pancreatic tumor-stromal interactions [60]. However, we focused on TGF-β signaling in TNBC progression and found that CAFs activated the TGF-β signaling pathway and promoted tumor growth. While SB431542, a TGF-β antagonist, inhibited the tumor growth promoted by CAFs, it did not inhibit tumor growth without CAFs. Taken together, these results suggest that TGF-β pathway regulated by CAFs is a molecular target of PFD.

PFD monotherapy at 50 mg/kg in mice (equivalent to the dose used in human) inhibited the CAF number significantly and tumor fibrosis and TGF-β signaling strongly, but had no effect on tumor growth or lung metastasis. Since TGF-β inhibitors can suppress primary tumor growth and metastasis *in vivo* [59, 61], it is possible that PFD monotherapy might inhibit cancer progression at a higher dose. Indeed, 500 mg/kg/day of PFD monotherapy suppresses the growth of pancreatic tumors transplanted with stellate cells orthotopically into mice [60]. Whether this effect occurs in other tumor types or at other doses has yet to be determined.

Doxorubicin has antitumor activities by the disruption of topoisomerase-II-mediated DNA repair and the generation of free radicals [62] and also activates TGF-β signaling (Figure 5D and [61]). In clinical treatment for breast cancer, doxorubicin (60 mg/m^2^) is administered intravenously every 3 weeks for 4 cycles [63, 64]. Since we observed that doxorubicin monotherapy inhibited primary tumor growth but not lung metastasis of TNBC, our data support the hypothesis that activation of TGF-β signaling by doxorubicin can lead to collagen accumulation but cannot suppress lung metastasis [21]. However, the combination therapy of doxorubicin and PFD inhibited primary tumor growth synergistically and lung metastasis significantly as seen previously for doxorubicin in combination with a competitive TGF-β RI inhibitor [61]. We suggest that simultaneous inhibition of TGF-β by PFD along with TGF-β activation as well as antitumor activities by doxorubicin may be important as mechanisms of synergistic effects of the combination therapy. Interestingly, chemotherapy-induced TGF-β signaling activation and TGF-β inhibitors prevent the development of drug-resistant cancer stem-like cells in TNBC [65]. TGF-β promotes breast cancer cell outgrowth from dormancy in metastatic sites, and our PDX models implicate TGF-β signaling activation in metastasis-initiating cells [20, 66], suggesting that combination therapy of doxorubicin and PFD may have additional inhibitory effects on metastasis-initiating cells.

Since the combination therapy of doxorubicin and PFD may have inhibitory effects on primary tumor growth and metastasis, it has great potential for a novel clinically applicable TNBC therapy that targets tumor-stromal interaction.

## MATERIALS AND METHODS

### Mouse transplantation models

All animal protocols were reviewed and approved by the UCSF IACUC. Mice were maintained under pathogen-free conditions in the UCSF barrier facility. PDX tumor tissues from TNBC patients were acquired from the laboratory of A. Welm [19] and engrafted in the mammary glands of immunodeficient NOD/SCID mice (Charles River Laboratories). After the engrafted tumors grew, they removed and cells separated for CAF culture.

We transplanted 4T1-GFP TNBC cells into cleared mammary fat pads of BALB/c mice (Simonsen Laboratories, Inc.). After three weeks, mammary tissues near the tumors were isolated, digested with collagenase I and IV and trypsin, and plated on dishes for culture. Cells grew in 2 weeks and GFP^−^ cells (CAFs) were isolated by flow cytometry to remove the contaminated 4T1-GFP tumor cells. 4T1 cells (1x10^4^) without or with the CAFs (2x10^4^) were injected in a 10-μl volume of 1:1 v/v Matrigel:DMEM/F12 medium into the inguinal mammary glands of BALB/c mice. Two dose protocols were used as indicated: 50 mg/kg or 100 mg/kg pirfenidone (Cipla Pharmaceuticals Ltd. Pirfenex) was orally administered two times a day. 4 mg/kg doxorubicin (LC Laboratories) or PBS was injected into the mouse tail vain on days 0 and 19 in the first protocol and on day 1 and 23 in the second protocol. Tumor volumes (mm^3^) were calculated using the formula: V = 0.52×W^2^×L. W=width (mm), L=length (mm).

### Cell culture

Pirfenidone (Sigma-Aldrich #P2166) was used for *in vitro* experiments. Tumor specimens of breast cancer patients from UCSF Medical Center (courtesy of Dr. H. Rugo) and xenograft tumors were digested with collagenase I and IV and trypsin, and plated on dishes for CAF culture. CAFs were grown in ACL4 + 5% FBS medium [67]. The cells were stained with trypan blue to detect live and dead cells.

MDA-MB231 cells were obtained from UCSF Cell Culture Facility. Murine TNBC 4T1 cell line was obtained from the American Type Culture Collection (ATCC) and labeled with GFP for transplantation. The cells were fixed with 4% PFA for immunocytochemistry.

### Histology

Tumor and lung tissues were fixed in 4% PFA overnight and paraffin processed. We cut 5-μm sections from paraffin-embedded blocks. Standard hematoxylin and eosin (H&E) staining was performed for routine histology. Picrosirius red staining was performed as previously described and fibrillar collagen visualized using crossed polarizers [54, 68].

Immunohistochemistry was performed as described below.

### 3D assay

Mammary glands of FVB/n mice were digested with collagenase. Organoids were collected by brief centrifugation and digested with trypsin to dissociate into single mammary epithelial cells (MECs) [69, 70]. The single MECs or 4T1 cells (5 x 10^4^ per well) were aggregated overnight on ultralow attachment plates (Corning). The aggregated MECs or the aggregated 4T1 cells + CAFs (2.5 x 10^4^ per well) were embedded into growth-factor-reduced Matrigel (BD Biosciences) and grown in serum-free media supplemented with insulin-transferrin (Invitrogen) and 2.5nM FGF2 (Sigma) as previously described [71]. SB431542 (Sigma-Aldrich), a potent and selective inhibitor of transforming growth factor-β (TGF-β) superfamily type I activin receptor-like kinase (ALK) receptors, and pirfenidone (P2166) were used for 3D assay. 3D Matrigel was fixed with methanol/acetone at −20°C and embedded into OCT for frozen sections.

### Immunofluorescence

Immunofluorescence of fixed paraffin-embedded tissue sections, fixed cells and frozen sections of fixed cultures in Matrigel was performed using the following antibodies at the indicated concentrations: phospho-SMAD2 (Cell Signaling #3101, 1:50), phospho-SMAD3 (Cell Signaling #9520, 1:50), TGF-β1 (Santa Cruz Biotechnology #sc146, 1:50), pan-cytokeratin (Sigma-Aldrich #C2562, 1:500), vimentin (Sigma #V5255, 1:200), human specific vimentin [V9] (Abcam #ab8069, 1:100), fibroblast activation protein (FAP) alpha (Abcam #ab53066, 1:200), alpha-smooth muscle actin (α-SMA) Cy3 conjugate (Sigma #C6198, 1:250), collagen I (Novus Biologicals #NB100-92161, 1:100), cleaved caspase-3 (Cell Signaling #9661, 1:200), phospho-histone H3 (Cell Signaling #9701, 1:100), goat anti-mouse IgM μ chain Cy3 conjugate (Jackson ImmunoResearch #115-166-075, 1:200), Alexa 488 anti-mouse, 488 anti-rabbit, 568 anti-rabbit, 647 anti-rabbit secondary antibodies (Molecular Probes A24920, A24922, A21069, A21245, 1:500). Nuclei were stained with DAPI (Vector Laboratories H-1200). Confocal microscopy was performed on a Nikon C1si confocal microscope.

### Flow cytometry analysis and cell sorting

TNBC xenograft tumors were digested with collagenase. Organoids were collected by brief centrifugation and digested with trypsin to dissociate into single cells. The cells were stained with antibodies against CD49f and EpCAM (eBioscience) for flow cytometry as described previously [72]. Cell sorting was performed on FACS Aria II (Becton Dickinson), and analysed using FACSDiva software (BD Biosciences).

### Cell viability assay

Cell viability was measured using the CellTiter MTT Assay according to the manufacturer's instructions (Promega). Sorted cells were plated in triplicate and incubated with PFD for 15 days, and attenuance at 590nm was read on sequential days using a plate reader (Bio-Rad).

### Lung metastasis analysis

To determine whether CAFs increased lung metastatic tumor frequency *in vivo*, lung tissue blocks were sectioned into 5-μm sections and stained by H&E. For each mouse analyzed, one section was scored for number of metastases per lobe. To test the effects of PFD on lung metastasis *in vivo*, lungs were harvested from each mouse, and then the weight was measured and the visible tumor number was counted.

### Statistical analysis

Statistical analysis was conducted using Prism 4 software (Graph Pad Software, Inc.). Statistical significance between two groups was calculated using Student's t test and P values lower than 0.05 were considered significant.

## SUPPLEMENTARY FIGURES


