# Supplementary material for: Targeting the cancer-associated fibroblasts as a treatment in triple-negative breast cancer
Source: Oncotarget. 2016 Oct 14;7(50):82889–901. doi: 10.18632/oncotarget.12658 (PMC5341254; doi:10.18632/oncotarget.12658)
Supplement: Supplementary file 1 [file oncotarget-07-82889-s001.pdf]

Targeting the cancer-associated fibroblasts as a treatment in triple-negative breast cancer

SUPPLEMENTARY FIGURES

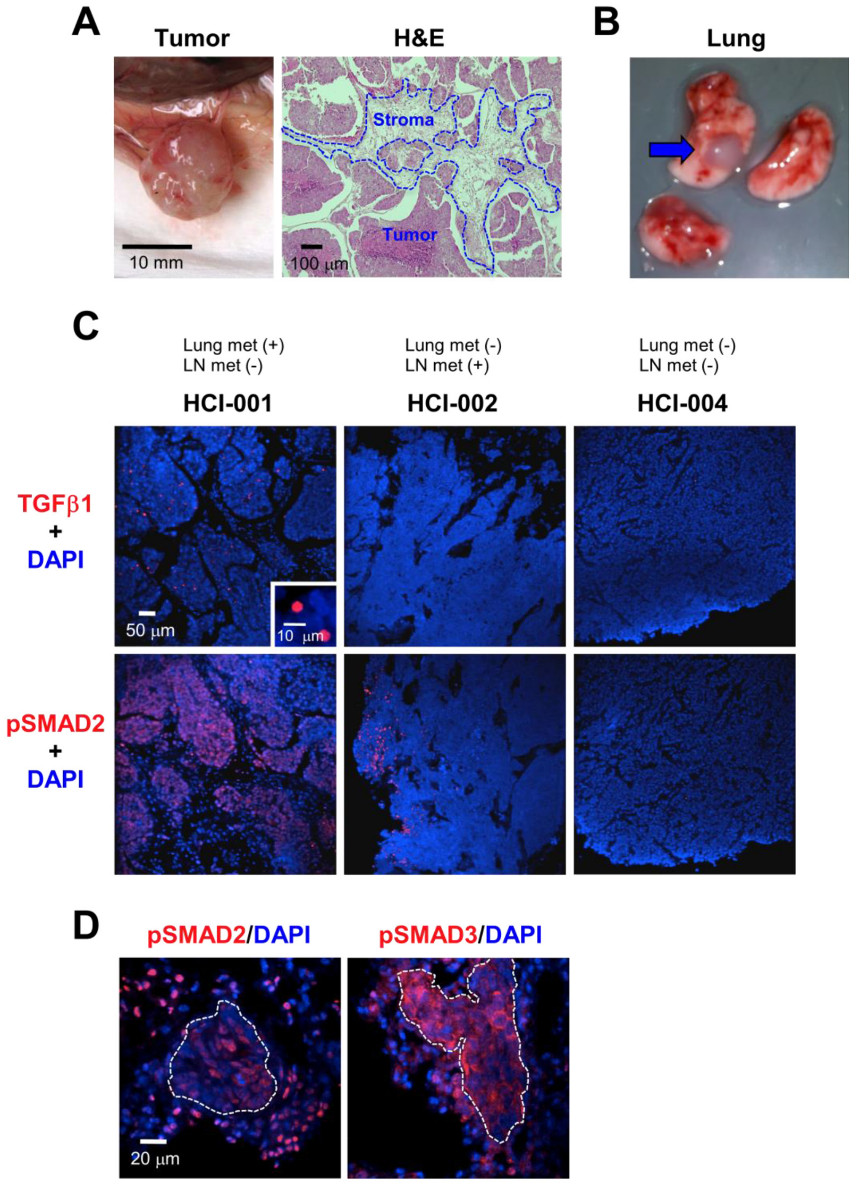

Supplementary Figure S1: PDX models of TNBC exhibit activated TGF-β signaling and lung metastasis. A. The human TNBC PDX models were engrafted in the mammary glands of immunodeficient NOD/SCID mice. A primary tumor of HCI-001 is shown (left panel). H&E staining of HCI-001 tumor is shown (right panel). **B.** Lung metastasis was detected visually in the HCI-001 PDX model. **C.** Three types of TNBC xenograft tumors were immunostained with anti-TGF-β1 (red on upper panel) and anti-phospho-SMAD2 (red on lower panel) antibodies. DAPI (blue) stained nuclei. TGF-β1 and phospho-SMAD2 were expressed in the primary tumors which metastasized to the lungs (HCI-001, left panel). Phospho-SMAD2 but not TGF-β1 was expressed in the primary tumors that metastasized to the lymph nodes (HCI-002, middle panel). Neither TGF-β1 and phospho-SMAD2 were expressed in the primary tumors, which metastasized to lungs nor lymph nodes (HCI-004, right panel). Levels of TGF-β1 signaling correlated with tumor aggressiveness. **D.** Lungs of the TNBC xenograft model (HCI-001) were immunostained with anti-phospho-SMAD2 (red on left panel) and anti-phospho-SMAD3 (red on right panel) antibodies. DAPI (blue) stained nuclei. Phospho-SMAD2 and phospho-SMAD3 were expressed in the lung metastatic tumors and the stroma around the large tumors.

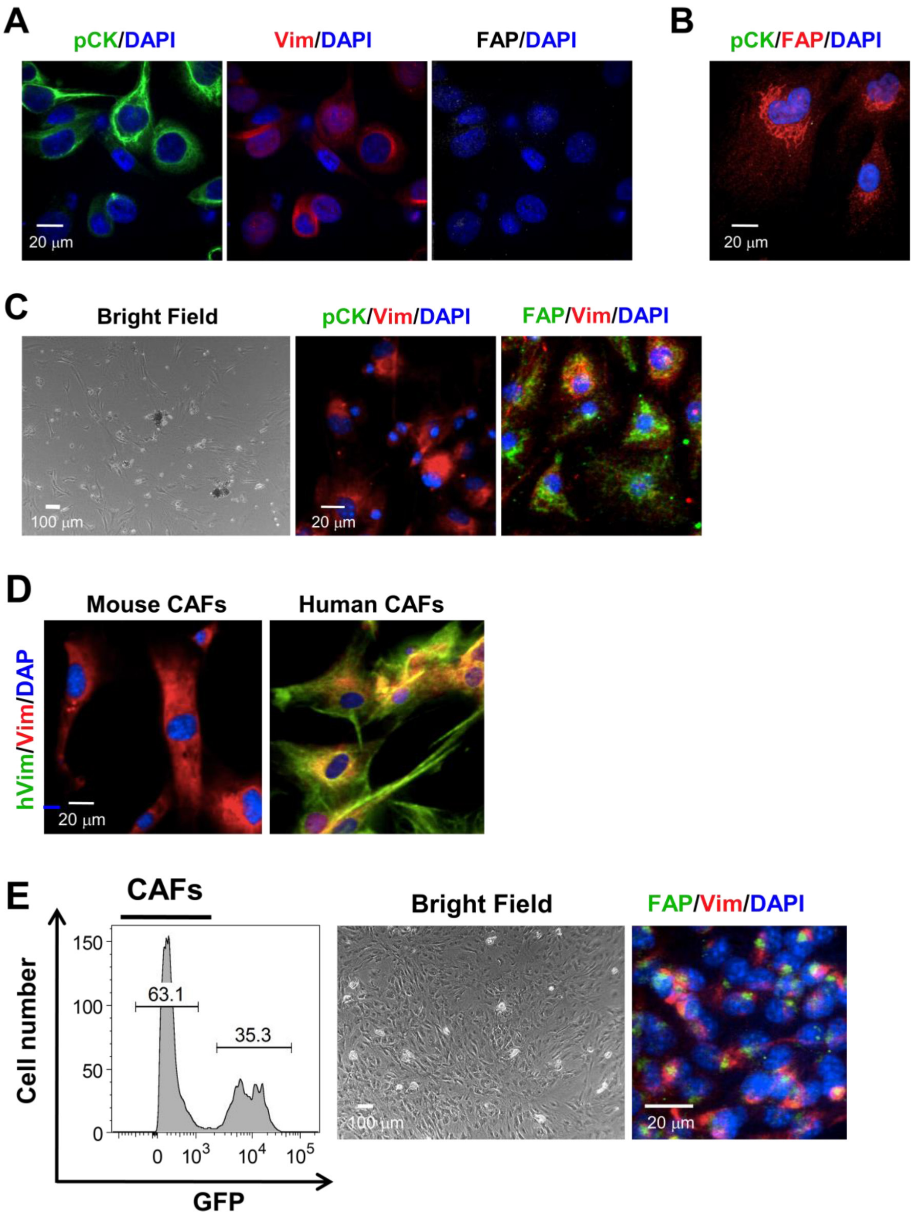

Supplementary Figure S2: Primary CAFs established from TNBC xenograft tumors and 4T1 TNBC homograft tumors. A. Immunofluorescence of MDA-MB231 cells was conducted by using anti-pan-cytokeratin (green in left panel), anti-vimentin (red in middle panel), and anti-fibroblast activation protein (FAP) (white in right panel) antibodies. DAPI (blue) stained nuclei. The cells were stained with anti-pan-cytokeratin and anti-vimentin antibodies, but not anti-FAP antibody. **B.** Immunofluorescence of cultured CAFs from fresh tumor specimens of breast cancer patients was conducted by using anti-pan-cytokeratin (green) and anti-FAP (red) antibodies. DAPI (blue) stained nuclei. CAFs were stained with anti-FAP antibody, but not anti-pan-cytokeratin antibody. **C.** We cultured CAFs derived from TNBC xenograft tumors (bright field, left panel). Immunofluorescence of cultured CAFs was conducted by using anti-pan-cytokeratin (green, middle panel), anti-FAP (green, right panel) and anti-Vimentin (red, middle and right panels) antibodies. DAPI (blue) stained nuclei. CAFs were stained with anti-vimentin and anti-FAP antibodies, but not anti-pan-cytokeratin antibody. **D.** Immunofluorescence of cultured CAFs from TNBC xenograft tumors and tumor specimens of breast cancer patients was conducted by using anti-human specific vimentin (green) and anti-vimentin (red) antibodies. DAPI (blue) stained nuclei. Since CAFs from the xenogrant were stained with anti-vimentin but not anti-human specific vimentin antibodies, they were derived from mice (left panel). On the other hand, the CAFs from breast cancer patients were stained with anti-human specific vimentin antibody (right panel). **E.** GFP- cells (CAFs) were isolated by FACS to remove the contaminated 4T1-GFP tumor cells (left panel). The CAFs were plated on 2-well chambers and grown in a 3% O_2 incubator (bright field, middle panel). Immunofluorescence of the cultured CAFs was conducted by using anti-vimentin (red) and anti-FAP (green) antibodies. DAPI (blue) stained nuclei. CAFs were stained with anti-vimentin and anti-FAP antibodies (right panel).

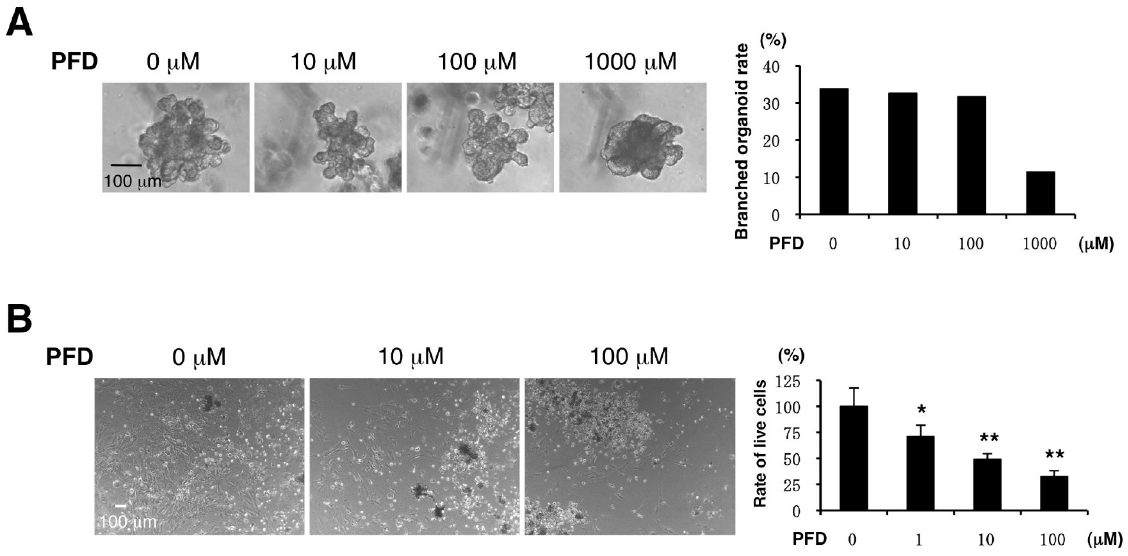

Supplementary Figure S3: PFD has inhibitory effects on cell viability in CAFs at the safe concentration. A. Normal mammary epithelial cells (MEC) from 7-week-old mice were aggregated overnight and 3D culture was conducted in Matrigel with FGF2-containing medium. High concentrations of PFD inhibited branching of mammary organoids (Day 5). Branched organoid ratio of branched organoids/total organoids. **B.** We cultured CAFs derived from TNBC xenograft tumors (HCI-001) and treated them with PFD in triplicate. Attached cells were stained with trypan blue on Day 4. Live cells decreased with the concentration of PFD. *$p<0.05$, **$p<0.01$ compared to the control condition.

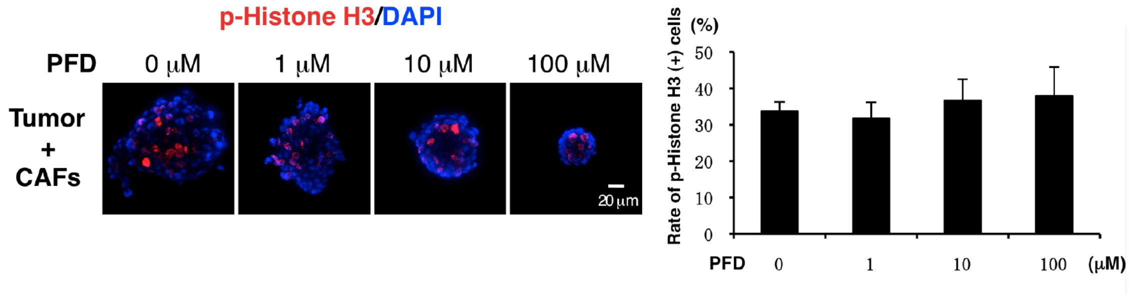

Supplementary Figure S4: PFD does not inhibit tumor cell mitosis. We conducted immunofluorescence of the 4T1 3D Matrigel cultures using anti-phospho-histone H3 (red) antibody. DAPI (blue) stains nuclei. PFD did not inhibit cell mitosis of 4T1 tumors.

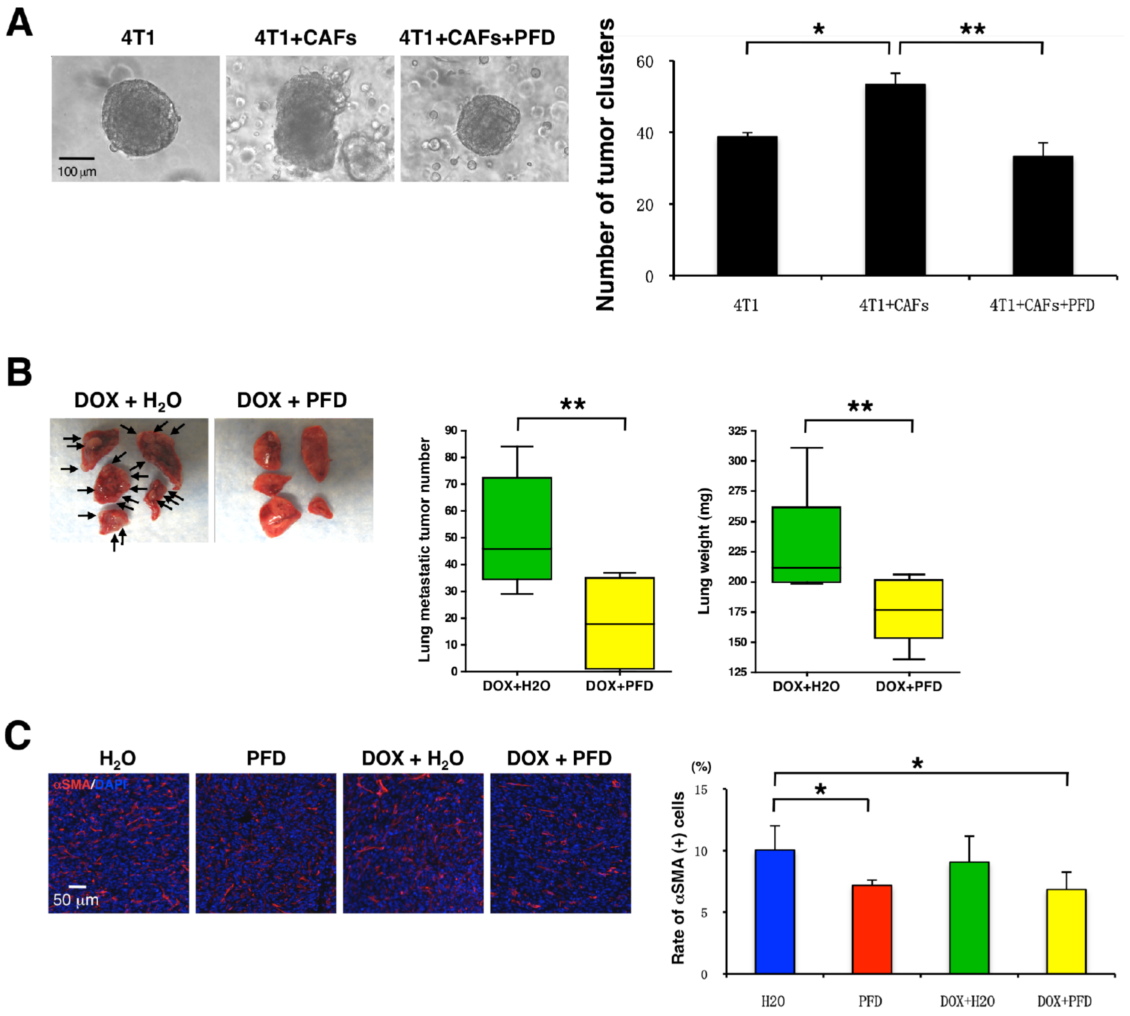

Supplementary Figure S5: Higher concentration of PFD increases inhibitory effects on lung metastasis in combination with doxorubicin. A. We conducted 3D co-culture assay of the 4T1-stimulated CAFs and aggregated 4T1 cells in Matrigel, and examined the effect of 100 μM PFD. CAFs increased the tumor cluster size and PFD inhibited the size increased by CAFs (left panel). Also, CAFs increased the number of tumor clusters and PFD decreased the tumor cluster number increased by CAFs (right panel). *p<0.001, **p<0.01. **B.** We transplanted 4T1 cells (2x10^4) and 4T1-stimulated CAFs (4x10^4) into mammary glands of BALB/c mice. Doxorubicin (4 mg/kg) was injected from the mouse tail vain on days 1 and 23 and PFD (100 mg/kg) or water was orally administered two times per day (n=5). Representative photographs showed that PFD inhibited the lung metastasis in combination with doxorubicin (Day 30) (left panel). Visible lung metastatic tumor numbers in five lobes were counted. PFD decreased the lung metastatic tumor number in combination with doxorubicin. *p<0.02 (middle left panel). Total lung weight was measured. PFD decreased tumor weight in combination with doxorubicin. **p<0.05 (right panel). **C.** α-SMA is also used as a CAF marker [1, 2]. To examine apoptosis in CAFs and tumor cells, primary tumors were immunostained with a mouse anti-α-SMA Cy3 antibody and a rabbit anti-cleaved caspase-3 antibodies. DAPI stained nuclei. Few apoptotic cells were detected in tumor cell regions and some apoptotic cells in necrotic regions. However, PFD did not promote apoptosis in α-SMA$^+$ CAFs and α-SMA$^-$ tumor cells (data not shown). On the other hand, PFD decreased α-SMA$^+$ CAFs (red) significantly. n=3, *p<0.05 (right panel). Representative photographs are shown (left panel).

REFERENCES

1. Barker HE, Bird D, Lang G and Erler JT. Tumor-secreted LOXL2 activates fibroblasts through FAK signaling. Mol Cancer Res. 2013; 11:1425-1436.
2. Yu Y, Xiao CH, Tan LD, Wang QS, Li XQ and Feng YM. Cancer-associated fibroblasts induce epithelial-mesenchymal transition of breast cancer cells through paracrine TGF-beta signalling. Br J Cancer. 2014; 110:724-732.
